# Glucose Decouples Intracellular Ca^2+^ Activity from Glucagon Secretion in Mouse Pancreatic Islet Alpha-Cells

**DOI:** 10.1371/journal.pone.0047084

**Published:** 2012-10-15

**Authors:** Sylvain J. Le Marchand, David W. Piston

**Affiliations:** Department of Molecular Physiology and Biophysics, Vanderbilt University, Nashville, Tennessee, United States of America; Joslin Diabetes Center, Harvard Medical School, United States of America

## Abstract

The mechanisms of glucagon secretion and its suppression by glucose are presently unknown. This study investigates the relationship between intracellular calcium levels ([Ca^2+^]*_i_*) and hormone secretion under low and high glucose conditions. We examined the effects of modulating ion channel activities on [Ca^2+^]*_i_* and hormone secretion from *ex vivo* mouse pancreatic islets. Glucagon-secreting α-cells were unambiguously identified by cell specific expression of fluorescent proteins. We found that activation of L-type voltage-gated calcium channels is critical for α-cell calcium oscillations and glucagon secretion at low glucose levels. Calcium channel activation depends on K_ATP_ channel activity but not on tetrodotoxin-sensitive Na^+^ channels. The use of glucagon secretagogues reveals a positive correlation between α-cell [Ca^2+^]*_i_* and secretion at low glucose levels. Glucose elevation suppresses glucagon secretion even after treatment with secretagogues. Importantly, this inhibition is not mediated by K_ATP_ channel activity or reduction in α-cell [Ca^2+^]*_i_*. Our results demonstrate that glucose uncouples the positive relationship between [Ca^2+^]*_i_* and secretory activity. We conclude that glucose suppression of glucagon secretion is not mediated by inactivation of calcium channels, but instead, it requires a calcium-independent inhibitory pathway.

## Introduction

Pancreatic islets respond to changes in blood glucose levels so that glucagon secretion from α-cells is maximal under hypoglycemic conditions (below 4 mM), whereas insulin is secreted from β-cells maximally at glucose levels greater than 8 mM [1], [2]. The primary function of glucagon is to prevent hypoglycemia by stimulating glucose output from the liver [3]. Once normoglycemia is reestablished, glucagon release is suppressed.

The molecular mechanisms leading to glucagon secretion and to its suppression by glucose are largely unknown [4]. Despite their opposite responses to glucose, α- and β-cells contain comparable secretory pathways: glucose transporters, the glycolytic enzyme glucokinase, ATP-sensitive K^+^ (K_ATP_) channels, high-voltage-gated calcium channels, and secretory granules [4]. In β-cells, breakdown of glucose generates disposable energy (increase in ATP to ADP ratio) that mediates the exocytosis of insulin granules via closure of K_ATP_ channels, plasma membrane depolarization, and calcium channel activation, respectively [5]. A similar K_ATP_- dependent depolarizing pathway appears to be present in α-cells, but its role in glucagon secretion, if any, is poorly understood. Various models have been proposed in which glucose overcomes this depolarizing pathway. In one model, low glucose concentrations set the α-cell membrane potential to a level that allows activation of voltage-gated sodium channels, which in turn leads to calcium channel activation and glucagon secretion. In this model, elevated glucose concentrations activate the K_ATP_- dependent depolarizing pathway, which then inactivates sodium channels, and suppresses glucagon secretion [6]–[8]. An alternate model speculates that paracrine inhibitors released from β-cells (e.g. insulin and zinc ions) suppress glucagon secretion by activation of α-cell K_ATP_ channels, membrane hyperpolarization, and inactivation of calcium channels [9], [10]. Although both models of glucagon suppression by glucose describe opposite effects on α-cell membrane polarization, they both rely on calcium channel inhibition. In contrast, we previously reported that α-cell calcium dynamics was not suppressed by glucose [1]. In the present study, we further investigate the relationship between α-cell [Ca^2+^]*_i_* and glucagon secretion at low glucose levels, and at inhibitory glucose concentrations. We examined the effects of modulating various ion channel activities that previous models have proposed to be important for α-cell function. We present a set of results acquired using islets from C57BL/6 mouse background performed under consistent conditions that allow comparison between different treatments. Using this approach, we measured the effects of glucose on [Ca^2+^]*_i_* and glucagon secretion from islets stimulated by glucagon secretagogues. Our results confirm a positive correlation between α-cell [Ca^2+^]*_i_* and glucagon secretion at low glucose levels, and indicate that greater glucose concentrations inhibit glucagon secretion independently from α-cell [Ca^2+^]*_i_* levels.

## Results

### Glucose Effects on Hormone Secretion from Perifused Islets

**Figure 1:**
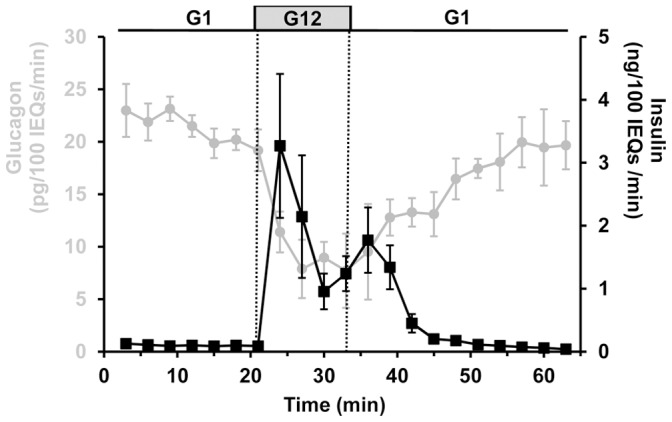
Glucose effects on hormone secretion from perifused islets. Isolated islets were exposed to 1 mM glucose for 30 minutes (from −30 to 0 min). Then, both glucagon and insulin responses (gray and black traces, respectively) were measured for 15 minutes at 1 mM glucose (G1). The perifusion was changed to 12 mM (G12) for 15 minutes, and then switched back to 1 mM. Experiment was repeated 3 times, 450 islets from 6 mice were used. Error bars represent the standard error of the mean. To compare the volume of islets with different diameters and volumes, individual islets were mathematically converted to standard islet equivalents (IEQs) with a diameter of 150 µm [44].

To establish the secretory dynamics of our system, we measured the time course of hormonal responses from perifused intact mouse islets exposed to a step-increase in D-glucose concentration (Fig. 1). By comparing the baseline secretion at 1 mM glucose with measurements obtained 15 to 18 minutes after glucose elevation to 12 mM, we found that glucose sharply reduces the rate of glucagon release by 64.8 ±16.4% (*p*<0.01). Meanwhile, insulin secretion from β-cells is strongly stimulated and exhibits a typical biphasic response composed of an acute first phase that lasts ∼10 minutes followed by a second phase plateau that starts at the nadir of the first phase [11]. A step-decrease in glucose from 12 to 1 mM restores maximal glucagon secretion and inhibits insulin secretion (Fig. 1). The recovery of glucagon secretion is much slower than its inhibition by glucose, consistent with previous reports [12]–[14].

### α-cell Responses to Modulation of High-voltage-gated Calcium Channel Activity

**Figure 2:**
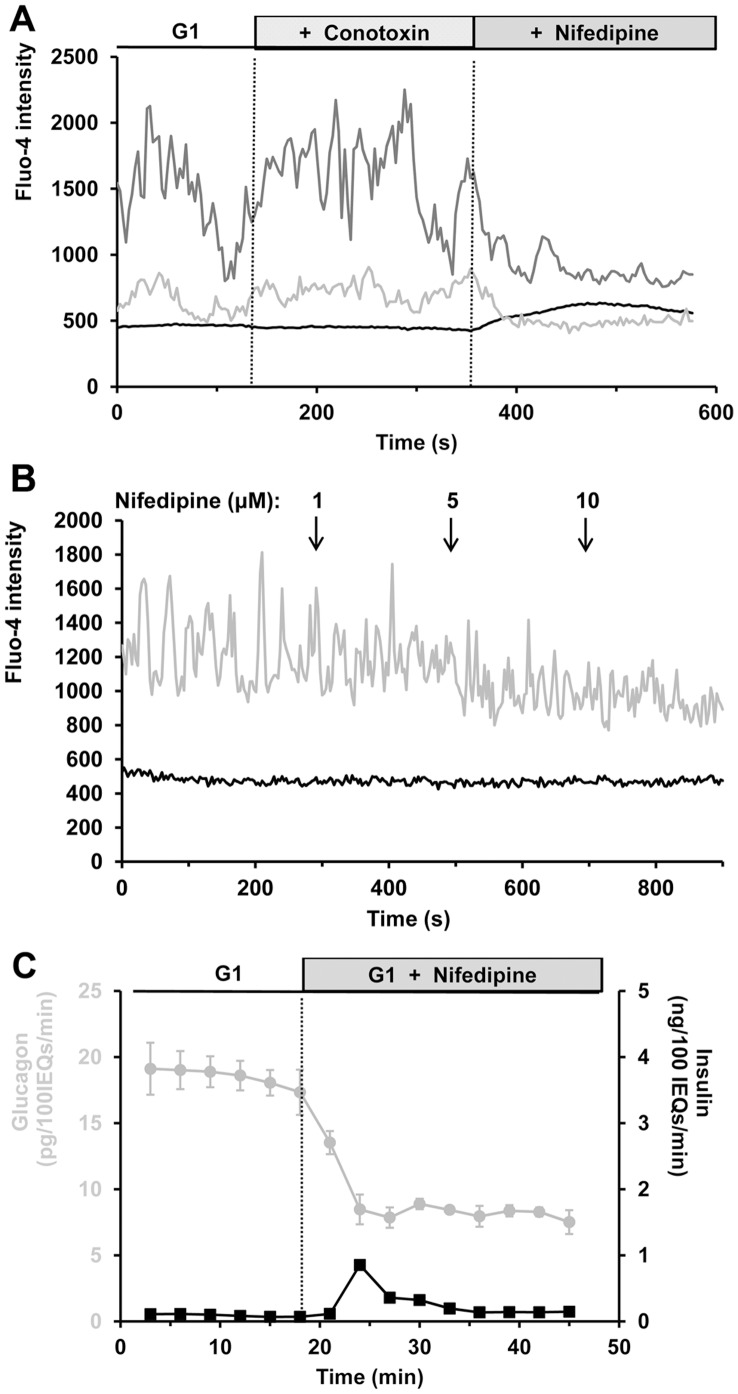
Effects of high-voltage-gated calcium channel inactivation on islet [Ca^2+^]*_i_* and hormone secretion. A, representative intracellular calcium responses to blockade of N- and L-type calcium channels. Gray traces represent α-cell [Ca^2+^]*_i_*, and black traces indicate β-cell [Ca^2+^]*_i_*. Fluo-4 intensity is expressed in arbitrary units. Calcium responses from two α-cells in the same islet are shown. N- and L-type channel inhibitors (1 µM ω-conotoxin and 20 µM nifedipine, respectively) were perifused. The figure is representative of 15 α-cells from 5 islets isolated from 3 mice. B, increasing concentrations of nifedipine were perifused at times indicated by the arrows. Nifedipine (≤ 10 µM) reduces calcium activity in α-cells without affecting β-cell [Ca^2+^]*_i_*. The figure is representative of 10 α-cells from 3 islets harvested from 3 mice. C, effects of nifedipine on hormone secretion from intact perifused islets. Islets were exposed to 1 mM glucose for 30 minutes (from −30 to 0 min). Glucagon and insulin responses (gray and black traces, respectively) were measured for 15 minutes at 1 mM glucose (G1), and 20 µM nifedipine was added to the perifusion medium for 30 minutes. Experiment was repeated 4 times, 600 islets from 8 mice were used. Error bars represent the standard error of the mean.

α-cells only constitute ∼15% of mouse islet cells [15]. As a result, it has been challenging to rigorously identify them in intact living islets. The transgenic expression of tdRFP in α-cells overcomes this limitation and is well-suited for Fluo-4 calcium imaging studies [1]. Nifedipine and ω-conotoxin were used to selectively block L- and N-type calcium channels, respectively [16]. In Figure 2A, a representative Fluo-4 experiment shows that ω-conotoxin at 1 µM does not affect either α- or β-cell [Ca^2+^]*_i_* at low glucose levels. Greater concentrations were also tested (up to 10 µM) but no response was observed. In contrast, nifedipine (20 µM) strongly inhibits α-cell calcium oscillations. On average, 20 µM nifedipine reduces α-cell Fluo-4 signal by 18.2 ±3.7% (n = 15, *p*<0.01). Lower concentrations of nifedipine also reduce the amplitude of calcium oscillations (Fig. 2B). Nifedipine inhibition of L-type calcium channels suppresses glucagon secretion by 55.0 ±3.7% (*p*<0.01) in islets perifused at low glucose levels (Fig. 2C). In contrast, ω-conotoxin did not affect glucagon secretion from intact islets in static incubation experiments at 1 mM glucose (0.69 ±0.29% of total cellular glucagon content secreted during 1 hour at 1 mM glucose, n = 5, vs. 0.62 ±0.25% in the presence of 1 µM ω-conotoxin, n = 5; *p* = 0.68).

We used a maximal level of 20 µM nifedipine in our experiments. Equal or greater concentrations of the drug have been utilized in islets [7], [17]. However, 20 µM nifedipine induces an increase in [Ca^2+^]*_i_* in some β-cells exposed to low glucose levels, whereas lower concentrations have no effect (Fig. 2A and 2B). This effect is possibly the result of non-specific inhibition of potassium channels by the drug that depolarizes β-cell membrane [18]. This depolarization would also explain the transient stimulation observed in the insulin response (Fig. 2C). This effect is unlikely to be responsible for the glucagon response because glucagon suppression occurs before the small rise in insulin release and persists after its termination.

### α-cell Responses to Modulation of Tetrodotoxin (TTX)-sensitive Na^+^ Channels

**Figure 3:**
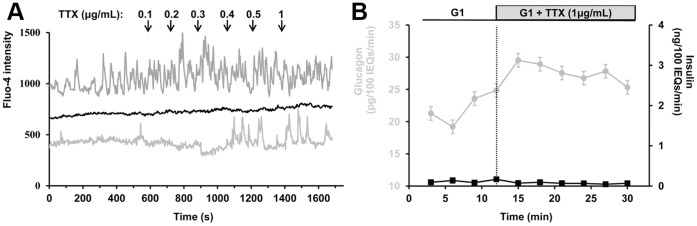
Effects of voltage-gated sodium channel inhibition on islet [Ca^2+^]*_i_* and hormone secretion. Gray and black traces represent α- and β-cells, respectively. A, representative intracellular calcium responses to tetrodotoxin (TTX) in an intact mouse islet perifused at 1 mM glucose. Increasing concentrations of TTX were perifused at times indicated by the arrows. TTX stimulates calcium activity in α-cells while having no noticeable effects on β-cells. Fluo-4 intensity is expressed in arbitrary units. The figure is representative of 18 α-cells analyzed from 6 islets harvested from 3 mice. B, effects of TTX on glucagon and insulin secretion from intact perifused islets. Isolated islets were exposed to 1 mM glucose for 30 minutes (from −30 to 0 min). Glucagon and insulin responses were measured for 9 minutes at 1 mM glucose (G1), and then TTX was perifused. Experiment was repeated 6 times, 900 islets from 12 mice were used. Error bars represent the standard error of the mean.

Activation of TTX-sensitive voltage-gated Na^+^ channels has been proposed to be involved in the activation of calcium channels at low glucose levels [6]–[8]. However, other electrophysiological studies failed to measure any residual sodium currents at physiological membrane potentials in α-cells [19], [20] but detected it in a subset of β-cells [19]. We therefore tested the effect of TTX on islets containing RFP-labeled α-cells loaded with Fluo-4. We applied TTX at low glucose levels to determine if inhibition of TTX-sensitive Na^+^ channels would inhibit α-cell [Ca^2+^]*_i_* oscillations. We did not observe any significant decrease in α-cell [Ca^2+^]*_i_* with TTX supplemented in the range of 0.1 to 1 µg/mL (i.e 0.3 to 3 µM) (Figure 3A). Although not statistically significant, TTX appears to induce a slight stimulatory effect on α-cells, as indicated by an acceleration in the frequency of [Ca^2+^]*_i_* oscillations (1.23±0.23 oscillation per minute vs. 1.47±0.29 when 1 µg/mL of TTX is applied, *p* = 0.18); while having no effect on β-cell [Ca^2+^]*_i_*. Islets exposed to 1 µg/mL TTX exhibit a 25.2 ±12.2% (*p*<0.05) increase in glucagon secretion (Fig. 3B), but TTX does not affect insulin secretion.

### α-cell Responses to Modulation of K_ATP_ Channel Activity

**Figure 4:**
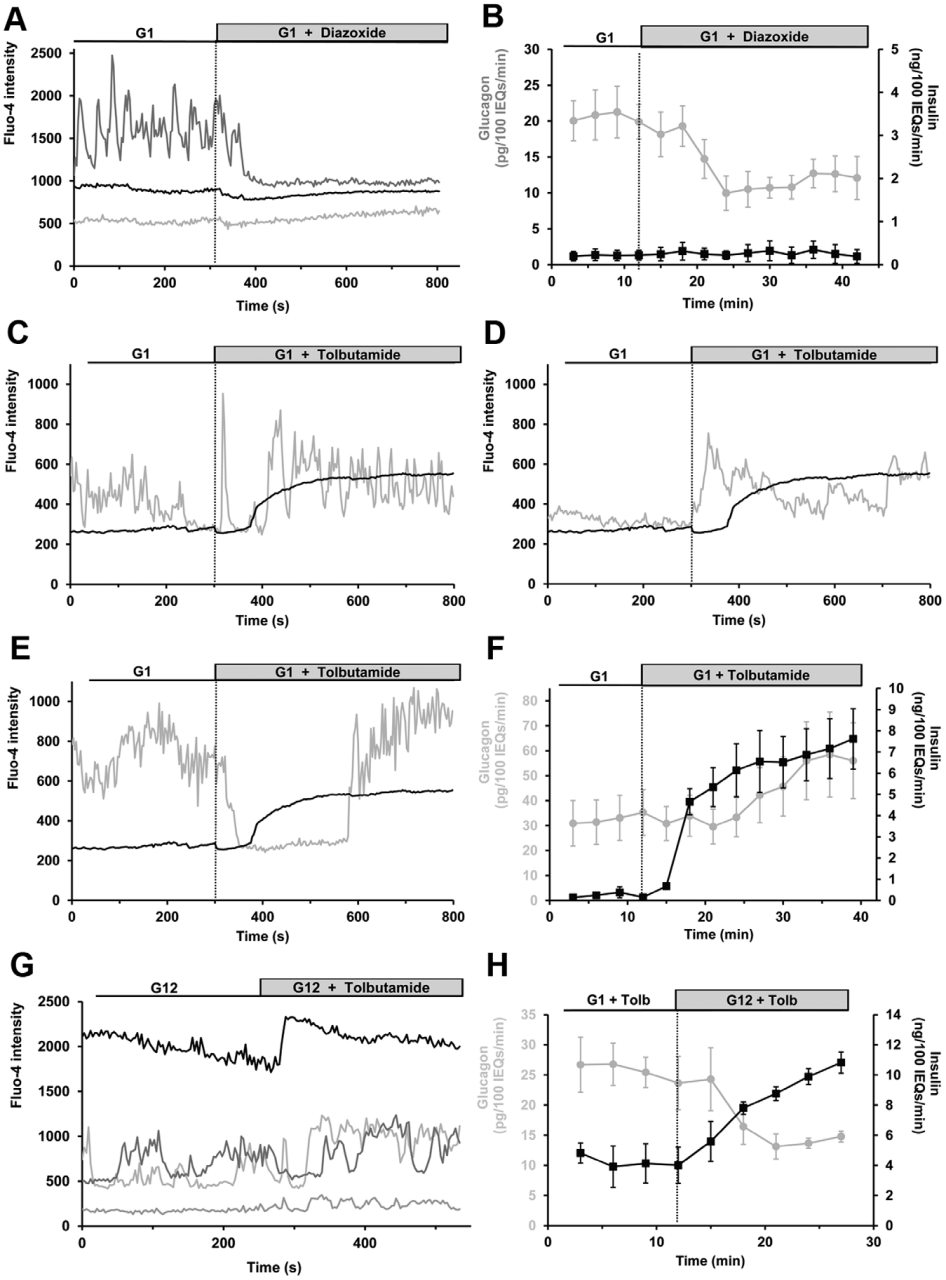
Effects of pharmacological modulation of K_ATP_ channels on islet [Ca^2+^]*_i_* and hormone secretion. Gray and black traces represent α- and β-cells, respectively. A, representative intracellular calcium responses to K_ATP_ channel activation by 100 µM diazoxide from an islet perifused at 1 mM (G1). Fluo-4 intensity is expressed in arbitrary units. The figure is representative of 25 α-cells analyzed from 7 islets harvested from 3 mice. B, Effect of diazoxide on hormone secretion from intact perifused islets. Isolated islets were exposed to 1 mM glucose for 30 minutes (from -30 to 0 min). Glucagon and insulin responses were measured for 12 minutes at 1 mM glucose, and diazoxide was perifused for 30 minutes at 100 µM. Experiment was repeated 3 times, 450 islets from 6 mice were used. Error bars represent the standard error of the mean. C, D, and E, representative Fluo-4 responses to K_ATP_ channel inhibition from an islet perifused at 1 mM glucose. The figure shows 3 different α-cells from the same islet exposed to 100 µM tolbutamide, and is representative of 34 α-cells analyzed from 10 islets harvested from 3 mice. F, glucagon and insulin responses were measured for 9 minutes at 1 mM glucose, and then tolbutamide was perifused at 100 µM. Experiment was repeated 6 times, 900 islets from 12 mice were used. G, representative Fluo-4 responses to 100 µM tolbutamide (TTX) in an intact islet perifused at 12 mM. H, glucagon and insulin secretion from islets perifused at 1 mM glucose and 100 µM tolbutamide for 15 minutes, then glucose concentration was increased to 12 mM. Experiment was repeated 3 times, 450 islets from 6 mice were used.

β-cell K_ATP_ channels couple cell metabolism to electrical activity and are therefore pivotal in triggering glucose-stimulated insulin secretion [5]. Expression of K_ATP_ channels has been reported in α-cells at similar or greater density compared to that in β-cells [20], [21], but their function in glucagon secretion remains poorly understood. [Ca^2+^]*_i_* was monitored by Fluo-4 fluorescence, and 100 µM diazoxide, a K_ATP_ channel activator, was perifused over the islets. Activation of K_ATP_ channels caused a strong reduction in the frequency and/or amplitude of calcium oscillations in 55% of oscillating α-cells (n = 20). Representative calcium responses are presented in Figure 4A. Diazoxide had no effect on the 20% of α-cells that were not oscillating, possibly because these cells were already too hyperpolarized to allow the activation of high-voltage-gated calcium channels. Overall, we found that diazoxide treatment at low glucose levels reduced the Fluo-4 signal by 22.6 ±5.2% (n = 25, *p*<0.01) in α-cells, whereas β-cell intensity was not affected (+9.8 ±9.7% in β-cells, n = 7 islets). Lower diazoxide concentrations (20 µM) were also found to inhibit calcium activity in 12 out of 15 oscillating α-cells (data not shown). Furthermore, diazoxide suppresses glucagon secretion by 47.8 ±13.0% (*p*<0.01) at 1 mM glucose, but does not affect insulin secretion (Fig. 4B). Because β-cell secretory activity is minimal under these conditions, α-cell inhibition by diazoxide is likely due to direct activation of α-cell K_ATP_ channels, and not to indirect paracrine effects.

A K_ATP_ channel blocker, tolbutamide (100 µM), was then applied to islets perifused at 1 mM glucose. We observed an increase in calcium activity in ∼45% (n = 34) of α-cells (Fig. 4C and 4D), but the effect of tolbutamide was heterogeneous from cell to cell. Some α-cells behave like β-cells and quickly respond by a strong rise in [Ca^2+^]*_i_*, whereas others responded more slowly. We noticed that ∼25% of α-cells exhibited transient inhibition in the frequency/amplitude of calcium oscillations for 5–10 minutes before recovering (Fig. 4E). In addition, tolbutamide had no significant effect on ∼25% of active α-cells, possibly because K_ATP_ channels were already closed in these cells. Finally, we observed an inhibition of calcium activity in ∼5% of the α-cells. On average, tolbutamide increased α-cell Fluo-4 signal by 36.9 ±11.5% (Fig. 4F) and glucagon secretion from islets by 62.8 ±26.7% (*p*<0.01). Under the same conditions, the Fluo-4 signal in β-cells increased by 140.8 ±25.0% (*p*<0.01), and insulin secretion was also elevated. The time-response to tolbutamide indicates that insulin release is quickly stimulated, whereas glucagon secretion is only enhanced after ∼15 minutes. This time-lag between insulin and glucagon responses corroborates our calcium measurements describing transient suppression of calcium activity in some α-cells (Fig. 4E).

We next sought to determine whether glucose could retain its inhibitory effect on α-cells that were stimulated by tolbutamide. We measured a 44.05 ±11.02% (*p*<0.01) decrease in the rate of glucagon secretion when 12 mM glucose was applied (Fig. 4H), while insulin secretion was increased from 4.13 ±1.30 to 10.36 ±0.44 ng/100IEQs/min. To test whether this glucagon suppression was mediated by a reduction in α-cell [Ca^2+^]*_i_*, we measured the effect of glucose on islets perifused with tolbutamide and found no significant change in Fluo-4 signal either in α- or in β-cells (4.61 ±10.97%, n = 12, and 0.11 ±17.51%, n = 5, respectively). A representative figure is presented in Fig. 4G.

### Effects of KCl- induced Depolarization of α-cells

**Figure 5:**
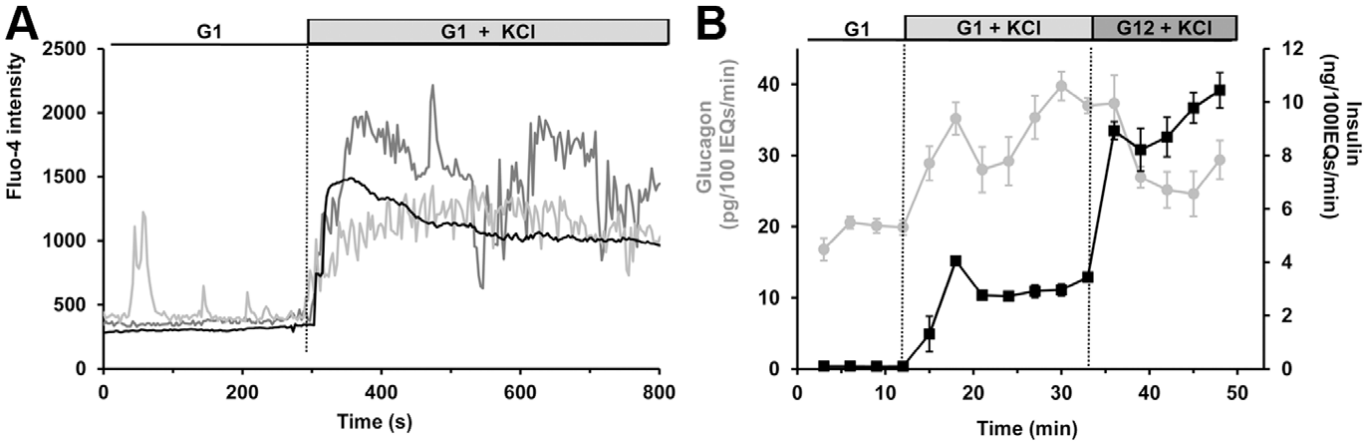
Effects of KCl on islet [Ca^2+^]*_i_* and hormone secretion. Gray and black traces represent α- and β-cells, respectively. A, intracellular calcium responses from an islet perifused at 1 mM followed by addition of 20 mM KCl. 2 α-cells from the same islet are presented. Fluo-4 intensity is expressed in arbitrary units. The figure is representative of 12 α-cells from 4 islets harvested from 3 mice. B, glucagon and insulin were measured for 9 minutes at 1 mM glucose (G1), and KCl was then perifused at 20 mM for 20 minutes, and glucose was added for 15 minutes at 12 mM (G12). Experiment was repeated 4 times, 600 islets from 8 mice were used. Error bars represent the standard error of the mean.

Fluo-4 measurements indicate that KCl application increases α-cell [Ca^2+^]*_i_* in both oscillating and non-oscillating cells, as shown in Fig. 5A. On average, Fluo-4 signal was enhanced by 76.8 ±23.0% in α-cells, whereas β-cell intensity was augmented by 132.9 ±28.9% (*p*<0.01, n = 14 α-cells from 5 islets isolated from 3 mice). Islet perifusion assays reveal that KCl stimulates glucagon secretion by 83.9 ±17.1% (*p*<0.01). KCl also increases the release of insulin (Fig. 5B). Application of 12 mM glucose reduces the rate of glucagon secretion by 29.57 ±9.13% (*p*<0.01), while stimulating insulin secretion from 2.73 ±0.17 to 9.23 ±0.43 ng/100IEQs/min. However, glucose addition did not change Fluo-4 signal in both α- and β-cells (8.22 ±12.31%, n = 7, and 6.72 ±13.09%, n = 4, respectively).

### Effects of Arginine on α-cells

**Figure 6:**
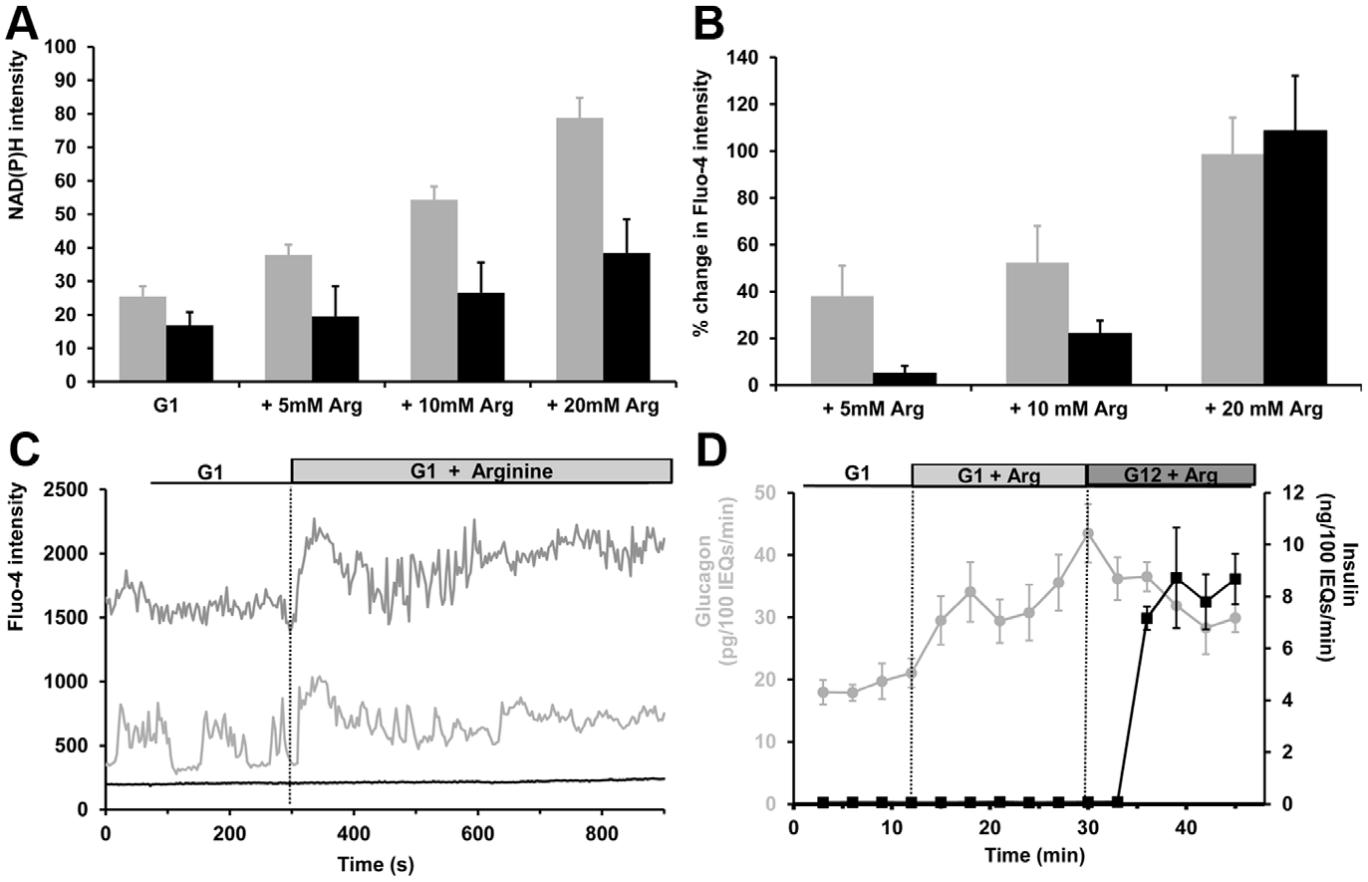
Effects of L-arginine on islet NAD(P)H, [Ca^2+^]*_i_*, and hormone secretion. Gray and black columns and traces represent α- and β-cells, respectively. A, arginine-dependent NAD(P)H responses from intact islets. Islets were perifused at 1 mM glucose (G1) and exposed to step-increases in arginine concentration. Data are normalized to minimal and maximal β-cell NAD(P)H obtained with FCCP and cyanide, respectively. The α-cell NAD(P)H changes to arginine (Arg) were statistically significant (*p*<0.01, 32 α-cells measured from 10 islets, 3 mice) and α-cell NAD(P)H intensity was different from β-cell intensity for each condition tested (*p*<0.01). Error bars indicate the standard error of the mean. B, averaged intracellular calcium responses to arginine. Data are expressed in percent change in Fluo-4 intensity compared to baseline at 1 mM glucose. α-cell responses to arginine were significant at all concentrations (*p*<0.01, n = 42) and β-cell responses were significant at 10 and 20 mM arginine (*p*<0.01, n = 11). C, intracellular calcium responses to 10 mM arginine from an islet perifused at 1 mM glucose (G1). 2 α-cells from the same islet are presented. Fluo-4 intensity is expressed in arbitrary units. The figure is representative of 11 α-cells from 4 islets. D, glucagon and insulin responses were measured for 9 minutes at 1 mM glucose, and then arginine was perifused at 10 mM for 18 minutes. Finally, glucose was added to the perifusion medium at 12 mM for 18 minutes. Experiment was repeated 4 times, 600 islets from 8 mice were used. Error bars represent the standard error of the mean.

The amino acid L-arginine is a potent glucagon secretagogue [22], but its mode of action has not been fully defined. Cell metabolism can be measured by changes in the autofluorescence of reduced pyridine nucleotides (NADH and NADPH), collectively referred to as NAD(P)H [23]. To determine the arginine-dependent NAD(P)H responses, we acquired the NAD(P)H intensities of islets at 1 mM glucose and compared them with those collected 15 to 30 minutes after arginine stimulation, when NAD(P)H signal has reached a plateau (data not shown). The islet NAD(P)H response to step-increases in arginine concentration was normalized to the minimal NADH redox state obtained with FCCP, and to the maximal signal with sodium cyanide (Fig. 6A), [1]. α-cells dose-dependently increase their NADH redox state with millimolar concentrations of arginine, to an extent similar to glucose [1]. This elevation in metabolic redox state indicates that arginine could activate a K_ATP_-dependent depolarizing pathway in this cell-type. In contrast, arginine was weakly metabolized in β-cells, as previously reported [24].

Arginine also dose-dependently elevates α-cell [Ca^2+^]*_i_* (Fig. 6B). We measure a 52.3 ±15.7% increase in Fluo-4 signal in α-cells in the presence of 10 mM arginine, compared to 22.1 ±5.4% in β-cells (16 α-cells from 5 islets, *p*<0.01 for both cell-types). Arginine quickly augments α-cell calcium activity (Fig. 6C), and this elevation in α-cell [Ca^2+^]*_i_* translates into increased rates of glucagon release (Fig.6D). 10 mM arginine stimulates glucagon output by 92.0 ±17.1% (*p*<0.01), whereas it has no effect on insulin secretion. The lack of insulin response at low glucose levels has been reported elsewhere [25].

Arginine-stimulated glucagon secretion is inhibited by glucose [25]. The rate of glucagon secretion was reduced by 26.4 ±9.8 (*p*<0.01) (Fig. 6D), while insulin secretion was strongly stimulated by glucose. Besides its suppressive effect on glucagon secretion, glucose slightly increased the arginine-stimulated α-cell Fluo-4 signal by 13.6 ±6.55% (*p*<0.05, n = 35 α-cells from 6 islets isolated from 3 mice). At the same time, 12 mM glucose increased β-cell Fluo-4 signal by 85.3 ±26.2% over than seen with arginine and 1 mM glucose.

## Discussion

The study of isolated islets with an α-cell label demonstrates that α-cell [Ca^2+^]*_i_* and glucagon secretion are closely related at low glucose levels. Inhibition of L-type voltage-gated calcium channels underlies calcium oscillations and secretory activity at low glucose levels (Fig. 2), supporting previous evidence [19], [26], [27]. However, blocking N-type channels was ineffective in inhibiting α-cells, contrary to some previous reports [7], [17], [28]. At least some of this difference may be due to species differences. Depolarizing agents such as tolbutamide and KCl augment both α-cell [Ca^2+^]*_i_* and glucagon secretion. Similarly, arginine stimulates glucagon secretion and increases α-cell [Ca^2+^]*_i_*. Since arginine is metabolized in α-cells, it likely activates a K_ATP_-dependent depolarizing pathway, although it can also increase the firing of action potentials by an electrogenic effect [8]. The rapid responses in [Ca^2+^]*_i_* and glucagon secretion after arginine treatment suggest that it predominantly stimulates secretion by a direct depolarizing effect mediated by its positive charge. In contrast, the metabolic responses obtained with glucose necessitate 5 to 10 minutes to be translated into an increase in [Ca^2+^]*_i_* [1].

We investigated the effect of TTX-sensitive voltage-gated sodium channels that have been proposed to set the α-cell membrane polarity to a level allowing activation of high-voltage gated calcium channels [6]–[8], [28]. In our hands, neither α-cell calcium oscillations nor glucagon secretion was inhibited by TTX (Fig. 3). In contrast, we observed a small increase in both α-cell calcium oscillatory activity and glucagon secretion. This may suggest that voltage-gated Na^+^ channels activate voltage-gated K^+^ channels involved in the inactivation of calcium channels [27], [29], but these data argue against a prominent role for voltage-gated Na^+^ channels in calcium channel activation and normal glucagon secretion.

In β-cells, glucose metabolism closes K_ATP_ channels and therefore depolarizes membrane potential from ∼−60 mV to ∼−35 mV [30]. L-type calcium channels start to open at membrane potential higher than −50 mV and are maximally activated between −20 mV and +10 mV [31]. Our results indicate that α-cell L-type calcium channels do not require the depolarizing effect of Na^+^ channels to be activated. This suggests that the α-cell membrane should be fairly depolarized at low glucose concentrations (>−50 mV), which is consistent with previous reports [27], [32]. This depolarized state likely originates from a higher metabolic state at low glucose concentrations, compared to β-cells (Fig. 6A, [1]), that would lead to greater ATP concentrations in α-cells, as reported in [33]. Thus, more α-cell K_ATP_ channels should be closed at low glucose levels, compared to β-cells, which is consistent with the reduced effect of tolbutamide on α-cell [Ca^2+^]*_i_* (Fig. 4). This hypothesis is further supported by the fact that α-cell K_ATP_ channels are more sensitive to ATP compared to β-cell K_ATP_ [20]. The observation that arginine and KCl elicit greater α-cell [Ca^2+^]*_i_* and secretion responses than does tolbutamide, suggests that they depolarize the α-cell membrane to a greater extent and thus activate more calcium channels. Interestingly, the glucagon response following arginine and KCl application is biphasic (Fig. 5B and 6D). The acute first phase may be the result of exocytosis of a readily releasable pool of glucagon-containing granules, whereas the acceleration in the rate of glucagon secretion during the second phase suggests that elevated [Ca^2+^]*_i_* promotes an amplifying pathway at low glucose levels, as seen in β-cells at greater glucose concentrations [5]. Activation of a non- K_ATP_-dependent amplifying pathway by glucose is observed in β-cells when islets are exposed to KCl and tolbutamide at high glucose levels (Fig. 4H and 5B). As a result, the rate of insulin secretion is increased whereas β-cell [Ca^2+^]*_i_* is not.

The role of α-cell K_ATP_ channels in glucagon secretion is controversial. Some studies have reported that blocking K_ATP_ channels would lead to calcium channel inactivation [6]–[8]. Our Fluo-4 imaging that assays all of the labeled α-cells reveals a subset in which tolbutamide suppresses calcium oscillations, but this inhibition was only transient (Fig. 4E). The different responses to tolbutamide (and diazoxide) application illustrate the importance of heterogeneities in the α-cell population, as some cells are in an excited state at low glucose levels while others are quiescent [1]. Electrophysiological experiments identify active cells, and those experiments find that only 7% of the cells examined are α-cells [17], even though >25% of the peripheral mouse islet cells should be α-cells. Contrary to findings based on the subset of α-cells that are active at any moment under low glucose conditions, we find that tolbutamide elevates α-cell [Ca^2+^]*_i_* in a majority of α-cells and stimulates glucagon secretion (Fig. 4). This positive effect is consistent with other reports [10], [21], [26], [28], [34] and suggests that K_ATP_ channels are active at low glucose levels, in contrast to some reports in which tolbutamide had no effect [35], [36]. Increased glucagon secretion in response to tolbutamide also challenges the paracrine model of glucagon suppression by glucose. Because β-cells are activated by tolbutamide, insulin and zinc are released and would be expected to inhibit glucagon secretion. Similarly, KCl depolarizes α-cell membranes and raises α-cell [Ca^2+^]*_i_*, while stimulating both glucagon and insulin secretion (Fig. 5, [37], [38]). In these cases, paracrine inhibitory products are apparently unable to overcome the stimulatory effect of tolbutamide and KCl on glucagon secretion, likely because they both force the α-cells into a depolarized state. Overall, the results obtained with tolbutamide, KCl, and arginine, indicate that membrane depolarization and calcium channel activation account for α-cell secretory activity at low glucose levels.

Activation of K_ATP_ channels by diazoxide increases the outward current of K^+^ and hyperpolarizes the α-cell membrane. The inhibitory effect of diazoxide on α-cell calcium activity (Fig. 4) suggests that membrane hyperpolarization inactivates L-type calcium channels, and thus suppresses glucagon secretion. α-cell K_ATP_ channel activity is therefore important for setting the membrane potential to a level that allows activation of calcium channels under low glucose conditions. Interestingly, both diazoxide and L-type channel blocker inhibit the secretion of glucagon to an extent similar to glucose (Fig. 1, 2C, and 4B). It is tempting to hypothesize that glucose mediates its inhibition by opening K_ATP_ channels and inactivating calcium channels, as proposed in [9], [10]. However, we have previously reported that α-cell calcium activity was not reduced by glucose [1]. We further illustrate this uncoupling between α-cell [Ca^2+^]*_i_* and secretion by showing that glucose suppresses arginine-stimulated glucagon secretion without a decrease in α-cell [Ca^2+^]*_i_* (Fig. 6). Similarly, glucose reduces the rate of glucagon secretion from islets stimulated by KCl and tolbutamide without affecting α-cell [Ca^2+^]*_i_*. In each of these cases, glucagon suppression by glucose is concomitant with an increase in β-cell secretory activity, so it is difficult to determine if suppression of glucagon results from direct effect of the sugar on α-cells or by an indirect paracrine inhibition from β-cells.

In summary, we propose that α-cell K_ATP_ channels are important at low glucose levels to create a fairly depolarized membrane potential that allows spontaneous activation of L-type calcium channels and exocytosis of glucagon-containing granules. Our results further show that K_ATP_ channels are not involved in glucose suppression of glucagon secretion. Instead, glucose inhibits secretion by a non-calcium-dependent pathway, which likely inhibits either granule mobilization to the membrane or glucagon exocytosis.

## Materials and Methods

### Materials

Fluo4-AM, fetal bovine serum, penicillin, streptomycin, Hanks balanced salt solution, phosphate buffer saline (PBS) and Roswell Park Memorial Institute (RPMI) 1640 medium were purchased from Invitrogen (Carlsbad, CA). Collagenase P was obtained from Roche (Basel, Switzerland) and tetrodotoxin from Tocris Bioscience (Ellisville, MO). Unless specified, all other products were purchased from Sigma-Aldrich (St. Louis, MO).

### Transgenic Mice and Islet Culture

All work with animals was conducted in compliance with the Vanderbilt University Institutional Animal Care and Use Committee (IACUC). Transgenic mice (C57BL/6 genetic background) that specifically express tandem-dimer Red Fluorescent Proteins (tdRFP) in α-cells have been described elsewhere [1]. Transgenic mice were identified by polymerase chain reaction (PCR) on mouse-tail DNA (Puregene Mouse Tail Kit, Gentra Systems, Minneapolis, MN). Oligonucleotide primers for Glucagon-Cre mice are: 5′-CCT CTA GGC TCA TTT GAC G-3′ (forward) and 5′-TCC ATG GTG ATA CAA GGG AC-3′ (reverse). ROSA26-tdRFP mice are identified with ROSA26 primers, as described in [39]. Islets were isolated and cultured as previously described [1]. Islets were cultured overnight for the secretion assays, and up to 3 days for imaging studies.

### Live-cell Imaging

Islets were imaged in a microfluidic device placed on the microscope stage, in a temperature controlled chamber at 37°C and 5% CO_2_. The microfluidic chip holds the living islet stable for imaging and allows rapid reagent changes. The fabrication of the device has been described elsewhere [40], [41]. Islets were studied in imaging solution (i.e. filtered aqueous solution containing: 125 mM NaCl, 5.7 mM KCl, 2.5 mM CaCl_2_ – 2H_2_O, 1.2 mM MgCl_2_, 10 mM HEPES and 0.1% bovine serum albumin, at pH7.4). For calcium imaging, islets were incubated with 5 µM of Fluo4-AM for one hour at 1 mM glucose. An increase in Fluo-4 intensity relates to greater [Ca^2+^]*_i_* and the response is a function of physiological free calcium concentrations [42]. After washing, islets were allowed to equilibrate on the microscope stage for 15 minutes. Fluo-4 was excited at 488 nm and its emission was recorded between 490 and 560 nm. Time-series images were acquired every 3 seconds. α-cells were localized thanks to the expression of tdRFP. TdRFP was excited at 561 nm and its fluorescence collected between 565 and 730 nm. We used a laser scanning microscope (LSM710, Carl Zeiss Inc., Thorwood, NY) with a Fluar 40x/1.3NA oil immersion lens (Zeiss). Confocal sections were obtained with a pinhole diameter set to 2 Airy units. Because β-cells constitute ∼80% of the cells in the islet [15], the Fluo-4 signal from non-tdRFP cells was considered to represent the average β-cell response. NAD(P)H measurements by two-photon excitation were performed as previously described [1].

### Measurements of Insulin and Glucagon Secretion from Perifused Islets

One day after isolation, islets were split into groups of 150 islets. Each group was placed into one of the individual glass chambers of a cell perfusion system for simultaneous study [43]. Islets were settled in each column on top of mesh filters with 25 µm pores to prevent islets from escaping into the effluent. Each experiment was preceded by a 30-minute stabilization period using a perifusion of 0.1% Dulbecco’s Modified Eagle Medium (DMEM) containing 0.1% BSA, 4.66 mM HEPES, 38.1 mM NaHCO_3_, 1 mM sodium pyruvate, 4 mM L-glutamine, and 0.015 ‰ phenol red, at pH7.4. Additional glucose and/or drugs were added to the perifusion medium as indicated in the text. A flow rate of 1 ml/min was used and samples were collected every 3 minutes by the Vanderbilt Islet Procurement and Analysis Core. Hormone secretion measurement was performed in duplicate by radio-immunoassays in the Vanderbilt Hormone Assay Core. Individual islets were mathematically converted to standard islet equivalents (IEQs) with a diameter of 150 µm [44]. 1h-static perifusion assays with ω-conotoxin were performed as described in [1].

### Data Analysis and Statistics

Image data were analyzed with Metamorph 7.6.1 (MDS Analytical Technologies, Downingtown, PA) and Excel 2007 (Microsoft, Redmond, WA) as previously described [1]. P values (two-tailed paired t-test) for glucagon measurements were obtained by comparing the baseline with measurements obtained 15 to 18 minutes after reagent change. Statistical analyses were performed by Prism 4 (GraphPad Software, La Jolla, CA).
